# Assessment of an LSDV-Vectored Vaccine for Heterologous Prime-Boost Immunizations against HIV

**DOI:** 10.3390/vaccines9111281

**Published:** 2021-11-05

**Authors:** Ros Chapman, Michiel van Diepen, Nicola Douglass, Shireen Galant, Mohamed Jaffer, Emmanuel Margolin, Phindile Ximba, Tandile Hermanus, Penny L. Moore, Anna-Lise Williamson

**Affiliations:** 1 Institute of Infectious Disease and Molecular Medicine, Faculty of Health Science, University of Cape Town, Cape Town 7925, South Africa; michiel.vandiepen@uct.ac.za (M.v.D.); niki.douglass@uct.ac.za (N.D.); shireen.galant@uct.ac.za (S.G.); emmanuel.margolin@uct.ac.za (E.M.); phindile.ximba@uct.ac.za (P.X.); anna-lise.williamson@uct.ac.za (A.-L.W.); 2 Division of Medical Virology, Department of Pathology, University of Cape Town, Cape Town 7925, South Africa; 3 Electron Microscope Unit, University of Cape Town, Rondebosch 7701, South Africa; mohamed.jaffer@uct.ac.za; 4 Centre for HIV and STIs, National Institute for Communicable Diseases of the National Health Laboratory Service, Johannesburg 2131, South Africa; TandileH@nicd.ac.za (T.H.); pennym@nicd.ac.za (P.L.M.); 5 Antibody Immunity Research Unit, Faculty of Health Sciences, University of the Witwatersrand, Johannesburg 2050, South Africa; 6 Centre for the AIDS Programme of Research in South Africa (CAPRISA), University of KwaZulu-Natal, Congella, Durban 4013, South Africa

**Keywords:** LSDV, lumpy skin disease, vaccine, HIV-1, vector, poxvirus, VLP

## Abstract

The modest protective effects of the RV144 HIV-1 vaccine trial have prompted the further exploration of improved poxvirus vector systems that can yield better immune responses and protection. In this study, a recombinant lumpy skin disease virus (LSDV) expressing HIV-1 CAP256.SU gp150 (Env) and a subtype C mosaic Gag was constructed (LSDVGC5) and compared to the equivalent recombinant modified vaccinia Ankara (MVAGC5). In vitro characterization confirmed that cells infected with recombinant LSDV produced Gag virus-like particles containing Env, and that Env expressed on the surface of the cells infected with LSDV was in a native-like conformation. This candidate HIV-1 vaccine (L) was tested in a rabbit model using different heterologous vaccination regimens, in combination with DNA (D) and MVA (M) vectors expressing the equivalent HIV-1 antigens. The four different vaccination regimens (DDMMLL, DDMLML, DDLMLM, and DDLLMM) all elicited high titers of binding and Tier 1A neutralizing antibodies (NAbs), and some regimens induced Tier 1B NAbs. Furthermore, two rabbits in the DDLMLM group developed low levels of autologous Tier 2 NAbs. The humoral immune responses elicited against HIV-1 Env by the recombinant LSDVGC5 were comparable to those induced by MVAGC5.

## 1. Introduction

The demand for new vaccines is ever increasing, be it for different pathogens or additional booster vaccines against a single pathogen. Despite the recent developments in vaccine technology [1], there remains a need for additional vaccine vectors, and live attenuated viral vectors are no exception [2]. The repeated use of the same live attenuated viral vector can result in anti-vector immunity, which could either be beneficial to the improvement of vaccine immunogenicity, or detrimental in the enhancement of viral infections [3]. Although low levels of antibodies can augment the uptake of a viral vector, high levels of pre-existing antibodies can reduce vaccine’s efficacy by the neutralization of the vector or inhibition of B cell responses [3]. Adenoviruses are widely used as live attenuated vaccine vectors. The human immunodeficiency virus 1 (HIV-1) STEP trial showed how pre-existing antibodies to adenovirus 5 (Ad5) resulted in the increase in HIV-1 infection in men vaccinated with the MRKAd5 HIV-1 Gag/Pol/Nef vaccine [4]. Since then, rare human adenovirus serotypes, as well as non-human primate adenoviruses, have been developed to overcome the problem of pre-existing antibodies; refinements to the adenovirus vector platform are also ongoing [5].

It has been forty years since the start of the HIV pandemic [6] and a vaccine for the disease remains unavailable. Despite decades of research into HIV-1 vaccine development, a vaccine inducing durable neutralizing immunity remains elusive [7,8,9]. Of the many different regimens tested in clinical trials, the DNA–MVA–protein regimen appears to be one of the most promising [10]. Recently, the recombinant MVA, expressing gp150, has been shown to induce a more durable antibody response than soluble gp120 protein following a DNA prime [11]. This study evaluates the HIV Env-specific antibody responses across ten different HIV vaccine regimens; MVA boosted longer lasting responses in comparison to protein boosts but of a lower magnitude [11]. In a comparison between DNA, MVA, and the vesicular stomatitis virus (VSV) as primes to a protein boost, MVA elicited higher binding and neutralizing antibodies to HIV Env than the other vectors in macaques [12].

Different vectors and adjuvants activate specific cellular pathways of immunogenicity, and specific B cell transcriptomic signatures have been associated with protection [11]. Likewise, different poxviruses exhibit different transcriptomic profiles [13]. The unique response elicited by the host to different vectors means that vectors can be selected according to the desired immune response. Poxvirus genera, historically, have been characterized by cross-neutralization and host range [14]; in particular, no cross-immunity has been shown between capripoxviruses and other poxvirus genera [15]. Lumpy skin disease virus (LSDV) is host-restricted to ruminants and, although there is some cross-neutralization of sheeppox virus (SPPV), this protection is only partial when cattle are vaccinated with SPPV and are subsequently challenged with LSDV [16]. Like capripoxviruses, orthopoxviruses have been shown to cross-neutralize within genera [17,18]; however, there is limited information on the cross-neutralization between different poxvirus genera. The neutralization of poxviruses is complex, with no single protein eliciting a neutralizing antibody response. There are six proteins required for a broad neutralization of the orthopoxviruses comprising vaccinia, variola, cowpox, and monkeypox; these are the mature virion (MV) proteins A27, L1, H3, and D8, and the extracellular virion (EV) surface proteins B5 and A33 [18]. In addition to vaccinia viruses (MVA and NYVAC), two avipoxviruses (canarypoxvirus (ALVAC) and fowlpoxvirus) have been extensively tested as vaccine vectors for HIV-1 vaccines [19]. There is no data available on the cross-neutralization of viruses within the *Avipoxvirus* genus, but whole genome sequence analysis has revealed the avipoxviruses to be highly diverse, genetically [20,21]. Poxviruses provide great potential for expanding the repertoire of vaccine vectors available for the future vaccine development for either humans or animals.

The application of veterinary research to human disease prevention is gaining momentum as part of a One Health approach to tackling global health problems [22]. The present study shows how a widely used live attenuated bovine vaccine, LSDV, can be used as a host-restricted vaccine vector for a human pathogen, HIV-1. Poxviruses are one of the best platforms for recombinant vaccine vectors. Recently, an Ebola vaccine based on the combination of an adenovirus and MVA vaccine vectors has been registered [23]. There are only a limited number of poxviruses that have been tested in human clinical trials; namely, vaccinia virus (VV), the attenuated host-restricted vaccinia viruses MVA and NYVAC, fowlpox virus, and ALVAC (based on the canarypox virus) [11,24,25,26]. Our group is investigating lumpy skin disease virus (LSDV) as a non-replicating vaccine vector as it is not able to replicate and complete the life cycle in non-ruminant hosts. LSDV, a member of the *Capripoxvirinae* genus of the *Poxviridae* family, causes significant morbidity in cattle in Africa and, more recently, in the Middle East and Europe [27]. The Neethling vaccine strain of LSDV has a proven safety profile, does not cause disease in immunosuppressed mice, and has already been investigated as a host-restricted vaccine vector for rabies and HIV-1 [28,29,30]. Encouragingly, vaccination with LSDV expressing rabies virus glycoprotein was able to protect mice from rabies challenge [30]. LSDV was also compared to MVA in both homologous and heterologous vaccine regimens as a potential HIV-1 vaccine vector in mice and macaques, eliciting high-magnitude, broad, and balanced CD4^+^ and CD8^+^ T cell responses. These responses were far greater than those observed in MVA or LSDV alone [28,29]. Both the LSDV and MVA HIV-1 vaccines expressed a polyprotein Grttn, consisting of Gag, RT, Tat, and Nef, but no Env and therefore antibody responses to Env were not tested in these previous studies.

With advances in HIV-1 vaccine research, vast improvements have been made in vaccine design to improve the induction of neutralizing antibodies [31,32]; our group has incorporated many of these features into making improved candidate vaccines against HIV-1 [33,34,35,36,37]. The CAP256 superinfecting viral envelope (CAP256.SU) was selected for use in this study as the patient from which the virus was isolated developed broadly neutralizing antibodies (bNAbs) [38]. In addition, this envelope was sensitive to several prototype bNAbs [39] and showed an enhanced reactivity to some bNAb precursors [40,41]. The Grttn polyprotein was replaced by HIV-1 CAP256.SU Env and truncated to gp150; the native leader sequence was replaced with the tissue plasminogen activator (TPA) leader peptide; the furin cleavage site was replaced with a flexible Gly-rich linker sequence (GGGGSGGGGSG); and a stabilizing I559P mutation was introduced into the gp41 region. In addition, a mosaic Gag gene was used so that virus-like particles (VLPs) could be produced. MVAGC5, expressing Gag^M^ and CAP256.SU gp150, was tested in rabbits in a DNA–MVA–protein regimen, with two doses of each of the vaccines. Tier1B and low levels of Tier 2 autologous neutralizing antibodies were produced by 4/5 rabbits [36]. The DDMMPP vaccine regimen is presently being tested in a non-human primate model [42]. The aim of this study is to construct and test the equivalent recombinant LSDV vaccine (LSDVGC5) to determine how it would compare to MVAGC5, and whether the neutralizing antibody response can be improved by the heterologous poxvirus vaccination. The vaccines are based on the HIV-1 subtype C, as this is the major subtype circulating in South Africa [43]. In addition to using improved HIV-1 antigen genes, improvements were also made to the construction of recombinant LSDV. Instead of using the Neethling LSDV vaccine strain as a parent virus backbone, the improved nLSDVSODis–UCT backbone was used [44] and recombination was targeted to an intergenic region between two highly conserved genes, LSDV ORFs 49 and 50. Previously, the HIV-1 Grttn gene was inserted into the ribonucleotide reductase gene [29], which would have caused the further attenuation of the LSDV. The previous construct contained the *Escherichia coli* xanthine-guanine phosphoribosyl transferase gene (Gpt) for positive metabolic selection, as well as the beta-glucuronidase (GUS) marker gene. The candidate vaccine made in the present study contained no metabolic selection gene and expressed the fluorescent protein, mCherry, instead of GUS.

The recombinant LSDV (L) expressing HIV-1 CAP256.SU gp150 and the HIV subtype C mosaic Gag (Gag^M^) was constructed and tested in a rabbit model using different heterologous vaccination regimens, in combination with DNA (D) and MVA (M). Groups of 5 rabbits were inoculated with 4 different vaccine regimens: DDMMLL; DDMLML; DDLMLM; and DDLLMM at 0, 4, 8, 12, 16, and 20 weeks. Binding and neutralizing antibody responses elicited by the different regimens were compared. LSDVGC5 was shown to elicit antibody responses comparable to MVAGC5, against HIV-1 Env.

## 2. Materials and Methods

### 2.1. Antibodies, Cell Lines, Media, and Reagents

Goat anti-HIV-1 gp160 (MRC ADP 72 408/5104); rabbit anti-HIV-1 p24 (Gag) (ARP 432); donkey anti-goat IgG Cy3 or FITC; and donkey anti-rabbit IgG Alexa 647 (Life Technologies, Oslo, Norway) were used for immunofluorescence assays. The goat anti-human IgG-FITC (Fc-specific) antibodies (ab 97224, abcam, Cambridge, UK) were used for live-cell staining. Goat anti-HIV-1 gp120 (Bio-Rad 5000–0557, Bio-Rad, Watford, UK); goat anti-HIV-1 p24 (Gag) (Bio-Rad 4999–9007, Bio-Rad, Watford, UK); and mouse monoclonal anti-goat/sheep IgG–AP GT34 (Sigma, Saint Louis, MO, USA) were used for Western blotting. The anti-HIV-1 Env human monoclonal antibodies (MAbs) PG9, PGT128, CAP256 VRC26.08, and VRC01 were expressed in FreeStyle 293F cells (Life Technologies, Oslo, Norway) using the PEIMAX transfection reagent (Polysciences, Warrington, PA, USA). The monoclonal antibodies were purified from cell-free supernatants after 6 days using protein A affinity chromatography [39].

MBDK and HeLa cells (supplied by ATCC) were grown in Dulbecco’s Modified Eagle Medium (DMEM) (high glucose), plus L-glutamine (Lonza), plus 10% of fetal calf serum, plus 10% of penicillin-streptomycin (Pen-Strep) (both from Gibco).

### 2.2. Design and Construction of DNA, MVA, and LSDV Vaccines Expressing Env and Gag

**Figure 1:**
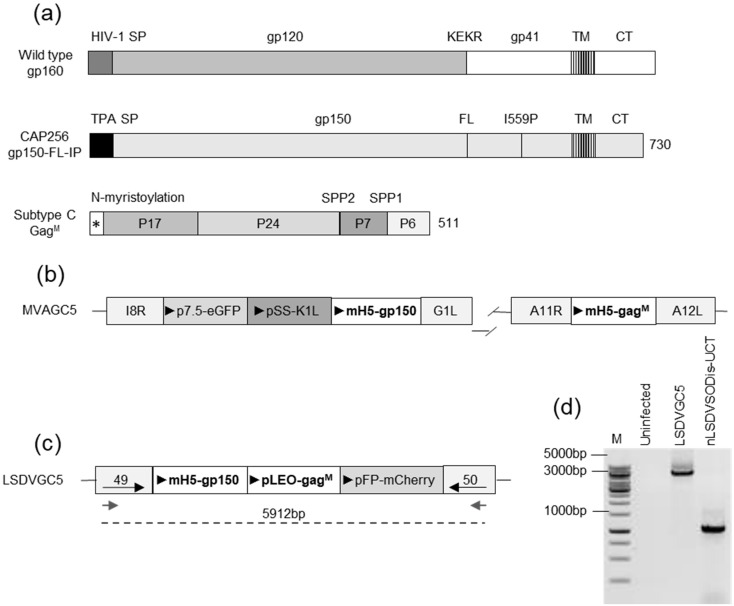
The design of vaccines. (**a**) The schematic representations of gene coding sequences for the HIV wild-type gp160 envelope, the gp150-FL-IP envelope immunogen, and the subtype C mosaic Gag (Gag^M^). The native signal sequence (HIV-1 SP) was replaced with the tissue plasminogen activator signal sequence (TPA SP) for the gp150 antigen, the furin cleavage site (KEKR) was replaced with a flexible linker (FL), and an I559P mutation was included. The gp150 protein was truncated at amino acid 730. TM—transmembrane domain and CT—cytoplasmic tail. No additional modifications were made to the subtype C mosaic Gag [46], *—N-myristoylation. (**b**) A diagram showing the design of MVAGC5. The expression cassette containing the gp150 was inserted between ORFs I8R and G1L and the Gag^M^ between ORFs A11R and A12L. (**c**) A diagram showing the design of LSDVGC5. An expression cassette containing gp150 and Gag^M^ was inserted between ORFs 49 and 50. The gray arrows indicate the primers used for PCR and the dotted line indicates the fragment amplified by PCR. (**d**) The PCR confirmation of LSDVGC5. DNA was extracted from MBDK cells infected with LSDVGC5 and subjected to PCR. Fragments were separated by agarose gel electrophoresis. M = GeneRuler 1kb DNA ladder (Thermo Fisher Scientific, Waltham, MA, USA).

The sequence of the CAP256.SU gp160 (clone CAP256.206sp.032.C9) has been previously described (GenBank: KF241776.1) [45]. The Env sequence was modified, as previously described [36]. As shown in Figure 1, the native leader sequence was replaced with the tissue plasminogen activator (TPA) leader peptide, the furin cleavage site was replaced with a flexible linker (FL) consisting of GGGGSGGGGSG, and an isoleucine was mutated to proline (I559P) in the gp41 region.

As previously described, the mammalian expression plasmid pTHPcapR [47] was used to construct DNA vaccines expressing the gp150; pMExT CAP256 gp150-FL-IP [36]; and subtype C mosaic Gag, pTJDNA4 [33]. In this study, the DNA vaccines pMExT CAP256 gp150-FL-IP and pTJDNA4 were administered together and are referred to as DNAGC5. All the DNA vaccines were synthesized using GenScript (Hong Kong).

MVAGC5 was constructed as previously described and shown in Figure 1b [36]. The expression cassette containing the gp150 was inserted between the I8R and G1L open reading frames (ORFs), and the gag^M^ gene was inserted between the ORFs A11R and A12L; both genes were placed under the control of the mH5 promoter.

The recombinant LSDV (LSDVGC5) was constructed using nLSDVSODis–UCT as a vector backbone [44]. For the construction of LSDVGC5, the green fluorescing LSDV(SODis)BEFV-Gb [48] was used as a parent virus so that the gene cassette between ORFs 49 and 50 could be replaced with a gene cassette, including the mCherry gene, which encodes a red fluorescing protein. A transfer vector was constructed in which the gene cassette—containing TPA-gp150-FL-IP under the control of the VACV mH5 promoter, gag^M^ under the control of the pLEO promoter, and the fluorescent marker mCherry under the control of the modified fowl poxvirus promoter [49]—was placed in between flanking sequences from the LSDV 49–50 locus (Figure 1b) [50]. Primary lamb tests cells were infected with LSDV(SODis)BEFV-Gb and transfected with the transfer vector. Three days after the infection and transfection, cells were freeze–thawed, and the viral lysate was passaged in the MBDK cells. Single, red fluorescing foci were repeatedly picked and passaged in the MBDK cells until no parent virus (green fluorescent foci) was visible. A single focus isolated from a 96-well plate was used to prepare a large-scale stock of LSDVGC5 in hyperflasks. T75 flasks of MBDK cells were infected at an MOI of 0.005 and incubated at 37 °C for 2 h with gentle mixing every fifteen minutes. The contents of the flasks were then used to set up hyperflasks which were allowed to grow for 11 days until most of the cells had lifted. Three freeze–thaw cycles were performed, and the supernatant containing the virus was cleared by low-speed centrifugation. The remaining virus in the pellet was released by lysis with 0.1 mM Tris (pH 9), and the second supernatant was added to the previous one after another low-speed spin. The LSDV was concentrated by centrifugation through a 36% sucrose cushion and resuspended in PBS plus 10% of glycerol. The recombinant (LSDVGC5) was then aliquoted and stored at −80 °C for downstream usage. Titers were determined in the MBDK cells, in which mCherry-positive plaques were counted 72 h after the infection of a serial dilution range. High-titer stocks were screened for the correct integration of the targeting construct by PCR and sequencing (analyzed with CLC Main Workbench, Qiagen). The expression of the mosaic Gag and Env was verified by Western blot analysis and immunofluorescence.

### 2.3. PCR Confirmation of the LSDV Recombinant

The insertion of the foreign gene cassette between LSDV ORFs 49 and 50 was confirmed by polymerase chain reaction (PCR), followed by agarose gel electrophoresis and Sanger DNA sequencing of the amplicon. The primer sequences used were 5′-GAGTGAAGCCTGGAACAT-3′ (forward primer) and 5′-ACTCTATCGCATCTGGAAACT-3′ (reverse primer). These generated fragment sizes of 1329 bp for control virus nLSDVSODis–UCT and 5900 bp for LSDVGC5. The Phusion High-Fidelity enzyme was used with the HF Buffer (New England BioLabs, Ipswich, MA, USA). The following thermocycling parameters were used for all PCR reactions: initial denaturation at 98 °C for 5 min followed by 40 cycles of denaturation at 98 °C for 30 s, annealing at 56 °C for 30 s, extension at 72 °C for 6 min, and a final extension at 72 °C for 10 min. PCR products were separated on 0.8% agarose gels, containing 0.5 μg/mL ethidium-bromide, by electrophoresis in 1xTBE buffer.

### 2.4. Live-Cell Staining Using Human Monoclonal Anti-Env Antibodies

To assess the structural integrity of Env expressed by HeLa or MBDK cells infected with LSDVGC5, binding to the anti-Env human monoclonal antibodies PG9, PGT128, CAP256 VRC26.08, and VRC01 was tested as previously described [36]; however, the secondary antibody anti-human IgG FITC (ab 97224, abcam, Cambridge, UK) was used.

### 2.5. VLP Isolation and Characterization

T150 flasks of the MBDK cells were infected with the LSDV at an MOI of 0.5. After 48 h, VLPs were isolated as follows: the media was cleared with a low-speed spin (5 min at 275 g), the supernatant was removed and transferred to centrifuge tubes, and centrifugation was performed at 47,000× *g* for 90 min at 4 °C with no break. Pellets containing VLPs were reconstituted in 100 µL of PBS (pH 7.4). Freshly activated carbon grids were coated with VLP preparations, stained with uranyl acetate, imaged onto an FEI Tecnai F20 transmission electron microscope (Thermo Fisher (formerly FEI), Eindhoven, The Netherlands), and fitted with a DE-16 camera (Direct Electron, San Diego, CA, USA).

For the preparation of thin sections, the MBDK cells infected with the LSDV from the T150 flasks were collected by scraping, spun briefly, washed with PBS, and then fixed overnight in 2.5% of glutaraldehyde in PBS (4 °C). The fixed cells were then washed twice in PBS and resuspended in 2% low melting point agarose and processed as described earlier [33].

### 2.6. Rabbit Immunizations

Female New Zealand White rabbits were bred and housed in the animal facility of the Faculty of Health Sciences at the University of Stellenbosch. Groups of 5 rabbits were used in the experiment. The rabbits were 4 to 5 months old and over 2 kg in weight at the start of the experiment. All the animal procedures were approved by the UCT Animal Research Ethics Committee (reference UCT AEC 015–051 and 019–015) and performed by trained animal technologists. DNA, MVA, and LSDV vaccines were administered intramuscularly into the hind leg at 100 µg (100 µL of each), 10^8^pfu (500 µL), and 10^7^pfu (500 µL), respectively, using the Pharmajet^®^ Stratis (Pharmajet, Golden, CO, USA) device. The vaccination schedule is shown in Figure 5a.

### 2.7. Anti-Env Enzyme-Linked Immunosorbent Assays (ELISA) and HIV Neutralization Assays

Env binding antibody titers in the rabbit sera were determined, as previously described [36]. The standardized TZM-bl pseudovirus neutralization assay was used to determine neutralizing antibody titers, as described previously [36].

### 2.8. Statistical Analysis

All statistical analysis was performed using the GraphPad Prism 5.0 (San Diego, CA, USA). Both one-way and two-way ANOVA were performed with Bonferroni post hoc testing. A non-parametric two-tailed Mann–Whitney test was also used when comparing the differences between the two groups.

## 3. Results

### 3.1. Design and Construction of Vaccines

The DNA, MVA, and LSDV vaccines were designed to express a membrane-anchored gp150 (Env) with the aim that the co-expression with the mosaic Gag (Gag^M^) would lead to the incorporation of Env into Gag virus-like particles (VLPs). Previous work carried out by our group has shown that the presentation of Env on the surface of Gag VLPs leads to better neutralizing immune responses, when compared to the HIV-1 envelope protein alone [36].

The envelope sequence was modified as previously described [36] and shown in Figure 1a, (CAP256 gp150-FL-IP). The mammalian expression vector, pTHPCapR, containing the porcine circovirus enhancer element, which has been shown to give increased antigen expression and immunogenicity [47], was utilized for the DNA vaccines expressing Env and Gag (DNAGC5) [33,36]. The recombinant MVA vaccine, MVAGC5, contains the HIV-1 subtype C mosaic Gag gene (Gag^M^) inserted between the A11R and A12L ORFs and the env gene inserted between ORFs I8R and G1L, as shown in Figure 1b [36]. Both genes are under the control of the VACV mH5 promoter.

To construct LSDVGC5, a transfer vector was designed containing the HIV-1 envelope gp150 gene under the control of the mH5 promoter, the Gag^M^ gene under the control of the pLEO promoter, and the mCherry marker gene under the control of a modified fowl poxvirus promoter. This expression cassette was inserted between the highly conserved, convergent LSDV ORFs 49 and 50 (Figure 1c). The recombinant was confirmed to be correct by PCR (Figure 1d) and sequencing.

### 3.2. Expression of Env and Gag

**Figure 2:**
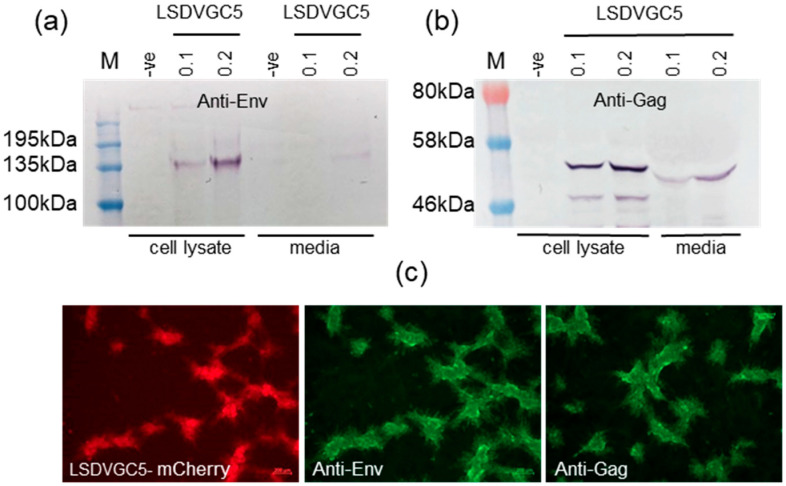
Confirmation of Env and Gag expression by cells infected with LSDVGC5. Western blots of cell lysates and media from MBDK cells infected with LSDVGC5. (**a**) Anti-Env; (**b**) anti-Gag; (**c**) and fluorescent images of BHK cells infected with LSDVGC5 (mCherry marker, red), and stained with anti-Env or anti-Gag antibodies followed by an anti-rabbit-Cy3 secondary antibody (green). -ve = uninfected cells, and 0.1 and 0.2 indicate the MOI of LSDVGC5 used.

The expression of the Env and Gag proteins in cells infected with the LSDV vaccine was confirmed by Western blotting and immunofluorescent staining (Figure 2). In vitro expression of Env and Gag has previously been confirmed for the DNA and MVA vaccines [34,36].

### 3.3. Quaternary Structure of Env Expressed In Vitro

**Figure 3:**
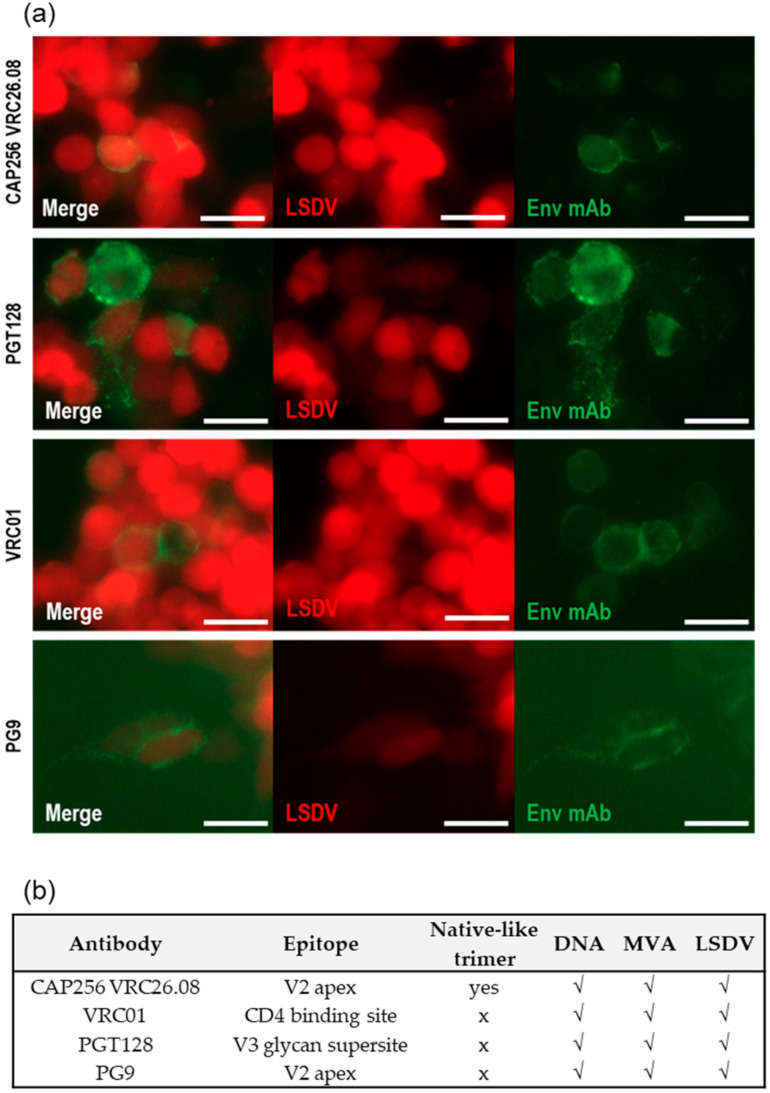
The characterization of Env expressed on the surface of cells transfected with DNA or infected with MVAGC5 and LSDVGC5 vaccines. (**a**) the live cell staining of cells infected with LSDVGC5, using MAbs CAP256 VRC26.08, PGT128, VRC01 (HeLa cells), or PG9 (MBDK cells). The infection with LSDV is visualized by mCherry expression (red). The binding of MAbs was detected with anti-human IgG-FITC (green). Scale bar = 50 µm. (**b**) Summary of MAbs to HIV Env that bound to cells transfected or infected with DNA, MVA, and LSDV vaccines expressing Env and Gag.

Live cell staining was carried out to assess the quaternary structure of the Env expressed on the surface of cells infected with the LSDV vaccine. All four human monoclonal antibodies (CAP256 VRC26.08, PGT128, VRC01, and PG9) tested, bound, to some extent, to the Env expressed by LSDVGC5 (Figure 3). The live cell staining of the cells transfected with the DNA vaccine or infected with the MVA vaccine was carried out previously with a larger panel of monoclonal antibodies [34,36]. A summary of the live cell staining results for the Env expressed by all three vaccines (DNA, MVA, and LSDV) is given in Figure 3b. CAP256 VRC26.08 and PG9 bound to a quaternary epitope on the V1/V2 of native trimers, indicating that at least some of the Env expressed by the three different vaccines was in a native-like conformation. The binding of PGT128 verified that the V3 glycan supersite was intact, and the binding of VRC01 confirmed the presence of the CD4 binding site.

### 3.4. Confirmation of Virus-like Particle Formation

**Figure 4:**
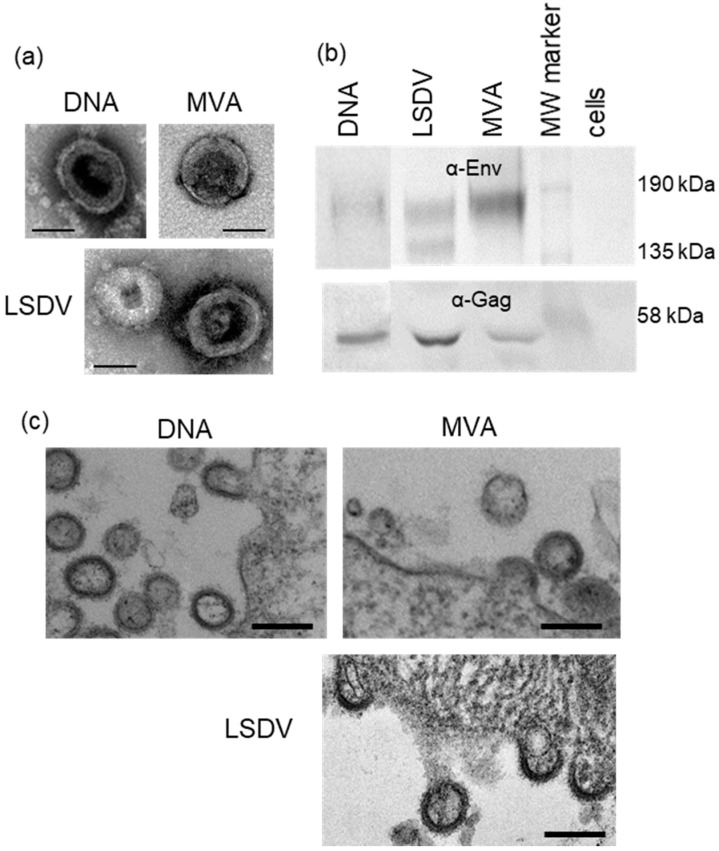
The in vitro formation of virus-like particles (VLPs) from cells transfected/infected with DNAGC5, MVAGC5, and LSDVGC5 vaccines. (**a**) The negative stain EM analysis of purified VLPs. Scale bar = 100 nm. (**b**) Western blotting of VLPs. Membranes were cut in half and the top half was probed with α-Env and the bottom was probed with α-Gag. (**c**) Electron micrographs of VLPs budding from cells transfected/infected with DNAGC5, MVAGC5, and LSDVGC5 vaccines. Scale bar = 200 nm.

All three vaccine candidates produced virus-like particles, as shown by transmission electron microscopy (TEM) of transfected/infected cells (Figure 4a,c), and previously confirmed for the MVA and DNA vaccines [34,36]. No VLPs were seen for uninfected/untransfected cells or cells infected with parental MVA or LSDV, confirming that VLP production was vaccine specific. Western blotting also showed that the VLPs contained both Gag and Env (Figure 4b).

### 3.5. Heterologous Vaccination with DNA and Two Different Poxvirus Vectors

**Figure 5:**
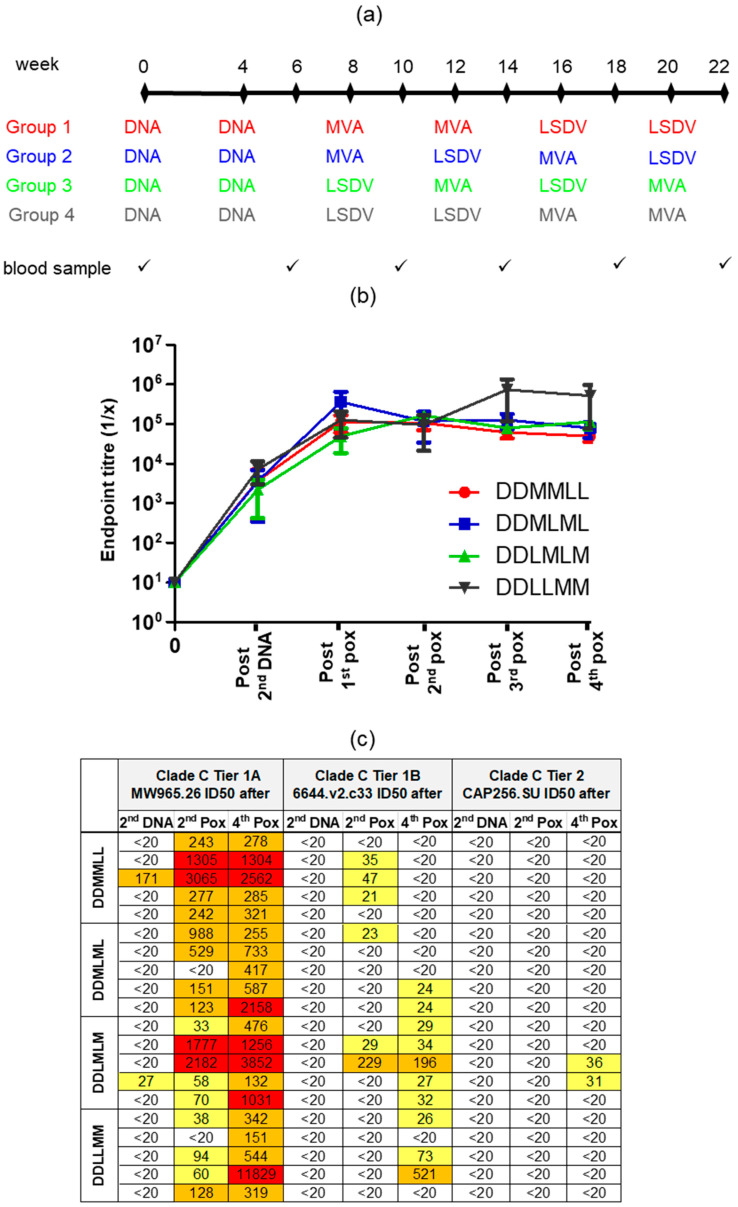
The antibody responses elicited in rabbits inoculated with different combinations of DNA, MVA, and LSDV vaccines. (**a**) Rabbit immunization protocol. All vaccines were administered using the Pharmajet Stratis^®^ device. (**b**) Time course showing the binding antibodies to Env (ELISA) in rabbit sera. When no binding was observed, the end point titer was plotted as 10. Data were plotted as the mean +/− SEM. (**c**) the neutralizing antibody titers in rabbit sera measured using the TZM-bl assay.

**Figure 6:**
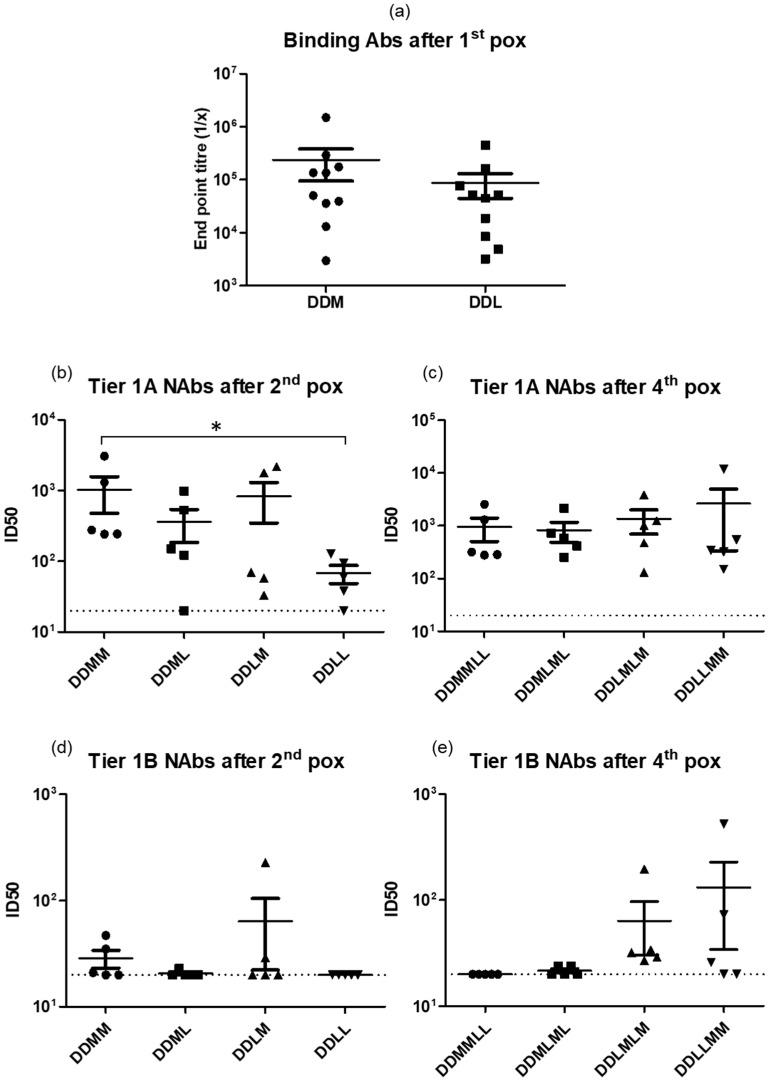
A comparison of antibody responses at different time points. (**a**) The binding antibody titers in rabbit sera after 2 DNA and a single poxvirus inoculation. Tier 1A and Tier 1B neutralizing antibody tires in rabbit sera after 2 DNA and 2 poxvirus inoculations ((**b**,**d**) respectively), and after 2 DNA and 4 poxvirus inoculations ((**c**,**e**) respectively). The dotted black line represents the assay detection limit (1/20 dilution). Data were plotted as the mean +/− SEM. * *p* > 0.008.

The use of LSDV and MVA vaccines in different combinations following two DNA primes was compared, as previous studies carried out in macaques by our group had shown that an MVA/LSDV heterologous prime boost elicited broader, higher magnitude T cell responses than either vector alone [28]. Four different vaccination regimens were compared, as shown in Figure 5a. All four regimens elicited similar levels of binding and Tier 1A neutralizing antibodies throughout the time course (Figure 5b,c and Figure 6a–c respectively). Two DNA followed by two MVA inoculations (DDMM) elicited significantly higher levels of Tier 1A Nabs compared to two DNA followed by two LSDV inoculations (DDLL) (*p* > 0.008), but no other differences were seen between the different groups (Figure 6b). The DDLMLM regimen appeared to elicit slightly better Tier 1B neutralizing antibodies in 5/5 rabbits, compared to 2/5 in the DDMLML regimen and 3/5 in the DDLLMM regimen, with slightly higher mean titers; however, this was not significant, probably due to the low numbers (Figure 6d,e). Similarly, only rabbits vaccinated with the DDLMLM regimen developed autologous CAP256.SU Tier 2 neutralizing antibodies. These results showed that the LSDV-vectored vaccine elicited very similar responses to MVAGC5, and LSDV could therefore be used as an additional vaccine vector for HIV-1 and other human pathogens.

## 4. Discussion

Several features make poxviruses ideal vectors for recombinant vaccines: poxviruses are able to accommodate relatively large pieces of foreign DNA; they are easy and cheap to manufacture; they are stable as freeze-dried preparations for long periods of time; they replicate in the cytoplasm, not the nucleus, and are therefore unlikely to get integrated into the host genome; and can induce long-lasting cellular and humoral immune responses. As discussed in a comprehensive review on recombinant capripoxviruses as vaccine vectors for delivering foreign antigens, recombinant LSDVs have been constructed expressing antigens against a variety of diseases, including rinderpest; peste des petits; Rift Valley fever; bluetongue; rabies; bovine ephemeral fever; foot and mouth disease; HIV; hydatidosis; and brucellosis [51]. Most of these recombinant LSDV vaccines induced effective immune protection when assessed. In this study, LSDV was evaluated as an HIV-1 vaccine vector and compared to MVA, expressing identical antigens.

Poxviruses, like most other viral vectors, do not perform optimally when used repeatedly in the same host due to vector immunity. Therefore, DNA is often used to prime the immune response which can be boosted by two subsequent MVA vaccinations. Studies have shown that a third MVA vaccination does not boost unless a substantial amount of time has passed [52]. This provides justification for the development of other poxvirus vectors, such as LSDV, for instances where additional boosts are necessary.

MVAGC5 had an improved design compared to the SAAVI MVA-C vaccine design [53] and included the use of different promoters for the Gag and Env genes; a replacement of the native Gag gene with the subtype C mosaic Gag antigen which forms virus-like particles; and the modification of Env by sequence changes to improve stability and transport to the cell surface [35]. A further improvement was based on the CAP256 superinfecting viral envelope (CAP256.SU) protein which had a flexible glycine linker and I559P mutation. In a previous study, heterologous prime-boost regimens with DNAGC5, MVAGC5, and protein, elicited autologous Tier 2 neutralizing antibodies (NAbs) and high-titer binding antibodies to the matching CAP256 Env and CAP256 V1V2 loop scaffold [36]. The presence of binding antibodies (ELISA), Tier 1 Nabs, and antibodies to V2 have been reported to correlate with the protection from SIV challenge [52,54]. The mosaic HIV subtype C Gag had been shown to induce T cell responses in mice [33]. T cell responses to Gag are associated with the control of HIV and it is therefore desirable to include Gag in HIV vaccines [32]. In line with the improved design of MVAGC5, LSDVGC5 (expressing HIV-1 CAP256.SU gp150 and the subtype C mosaic Gag) was constructed and shown to produce HIV-1 Gag VLPs containing Env. The conformation of the Env expressed on the surface of the cells infected with LSDVGC5 was characterized by live cell staining with bNAbs. The Env bound trimer-specific bNAb, CAP256 VRC26.08, indicating that a proportion of the Env was in a native-like trimeric conformation as shown for MVAGC5 [34,36]. However, the live staining, with MAb CAP256 VRC26.08, of cells infected with LSDVGC5 appeared to be slightly lower than that of the other MAbs that do not detect native-like trimers (Figure 3), possibly indicating that not all of the envelopes expressed had a native-like trimer structure. However, this was not quantified as this is difficult to do using fluorescent microscopy. This could explain why such low levels of autologous Tier 2 NAbs were elicited by the different vaccination regimens. Nevertheless, this is highly encouraging and confirms that some of the heterologously expressed antigens retain the desired conformation when expressed in vitro. Recapitulating bNAb epitopes is generally considered a hallmark of a good Env immunogen and, in many cases, this may require the extensive engineering of the antigen sequence. It is plausible that presenting Env in a membrane-bound context helped stabilize the protein. Clade C stable, native-like Env trimers are often more difficult to produce than other clades [55]. In this study, the only modifications made to the Env to improve stability and folding were to replace the furin cleavage site with a ten amino acid flexible linker and to include the I559P mutation in gp41. Further targeted modifications to the CAP256.SU Env sequence, to improve the structure and antigenicity, could increase the levels of stable, native-like CAP256.SU [56,57,58,59,60,61].

The role and importance of different types of antibodies in the protection against HIV infection is still not fully understood. In the RV144 trial, HIV-1-specific IgG3 distinguished two HIV-1 vaccine efficacy studies (RV144 and VAX003 clinical trials) and correlated with the decreased risk of HIV-1 infection [62]. Antibody-dependent cell-medicated cytotoxicity and antibody-dependent cell-mediated virus inhibition also play a role in viral control in macaque models [63] and clinical trials [64,65]. The immunogenicity of our candidate HIV-1 vaccine was tested in a rabbit model using different heterologous vaccination regimens, in combination with DNAGC5 and MVAGC5 expressing matching antigens: DDMMLL; DDMLML; DDLMLM; and DDLLMM at 0, 4, 8, 12, 16 and 20 weeks. Ten of the twenty rabbits in this study developed binding antibody responses to HIV Env after two DNAGC5 inoculations, whereas in previous studies carried out using this vaccine none of the rabbits developed binding antibodies at this time point [34,36]. The responding and non-responding animals were fairly evenly distributed between the different groups, with 2/5 animals responding in the first two groups and 3/5 in the second two groups. All rabbits developed high levels of binding antibodies after one poxvirus immunization, which substantially boosted the antibody response observed after the two DNA priming vaccines. These antibodies may have Fc receptor-mediated functions which is of interest, as non-neutralizing functional antibodies can play a critical role in the protection from HIV infection [66]. It should be noted that LSDVGC5 was given at a ten-fold lower dose than MVAGC5; this was because LSDV does not reach titers as high as MVA. Despite the difference in dose, there was no significant difference in the binding antibodies elicited by the two vaccines. This confirmed that both poxvirus vaccines could induce good immune responses and offer the prospect of dose sparing, which is highly desirable for a vaccine that requires widespread implementation, such as HIV.

The HIV-1 envelope has a number of different conformations with the closed conformation (Tier 2 and 3) being the one that is mostly present on circulating viruses. The Tier 1A conformation is regarded as an open conformation and Tier 1B as an intermediate conformation. [67]. It is a general consensus that the induction of HIV Tier 2 NAbs for the protection from HIV infection is desirable [8], although it is important not to discount the contribution of non-neutralizing antibodies and cellular responses. While neutralizing monoclonal antibodies can protect from SHIV challenge [68], there are also examples of vaccines which induce neutralizing antibodies that do not protect from challenge in animal models [69]. Some insight into protection via NAbs has been gained from the HVTN 703/HPTN 081 trial, in which participants received infusions of a NAb (VRC01) at a dose of either 10 or 30 mg per kilogram [70]. While the overall trial did not prevent HIV acquisition, the incidence of infection with VRC01-sensitive isolates was significantly different in VRC01 recipients vs. placebo (estimated prevention efficacy, 75.4%; 95% CI, 45.5 to 88.9), indicating the protection from VRC01 sensitive viruses. This implies that NAbs, at an appropriate titer, can protect from infection with sensitive viruses. Some differences were observed in Tier 1A NAbs titers at various time points. The lowest Tier 1A titers were observed in the DDLLMM group after two LSDVGC5 immunizations (DDLL). The clearest neutralization differences could be seen in the Tier 1B NAb responses elicited. All five rabbits in the DDLMLM group, three out of five rabbits from the DDLLMM group, and two out of five in the DDMLML group, developed low levels of Tier 1B NAbs. In the DDMMLL group, Tier 1B antibodies were detected after two MVAGC5 boosts, but not after the subsequent LSDVGC5 boosts. Three out of five rabbits from the DDLLMM had Tier 1B antibodies after the fourth poxvirus boost, but none were detected after the first two LSDVGC5 immunizations. All five rabbits had Tier 1B antibodies in the DDLMLM group, with two rabbits having antibodies after the first two immunizations and all five after four poxvirus immunizations. The Tier 2 NAb responses were disappointing; however, two out of five rabbits in the DDLMLM group developed low level autologous Tier 2 NAbs after the fourth poxvirus boost. As mentioned earlier, additional targeted sequence changes to improve the structure and antigenicity of the CAP256.SU Env used in this study can increase the levels of autologous Tier 2 NAbs elicited by the different vaccination regimens. In summary, the best antibody responses were observed in the DDLMLM group with the alternating combination of the two different poxvirus vectors. Increasing the time between homologous boosts might have resulted in a better response. Moreover, two MVAGC5 immunizations (DDMM) induced Tier 1B Nabs, which were not induced by LSDVGC5 (DDLL).

Poxviruses have been shown to differ with respect to the type of immune responses induced. HIV-specific humoral and cellular immune responses were compared in macaques vaccinated with NYVAC (orthopoxvirus) and ALVAC (avipoxvirus). Two doses of the poxvirus followed by two doses of the poxvirus plus gp120 protein were given to the animals [71]. NYVAC induced higher antibody responses to HIV Env and showed a trend towards eliciting higher cellular responses. An analysis of the transcriptome following infection of mice with LSDV, MVA, canary poxvirus, fowlpox virus, and two novel avipoxviruses showed that all six poxviruses induced distinct gene expression profiles. LSDV caused the most significant response in comparison to the other poxviruses, both in the magnitude and breadth of type 1 interferon responses and in the number of up-regulated genes involved in antigen processing and presentation pathways. CNPV and FWPV induced the up-regulation of the immunoglobulin gene expressing IgG3. Env IgG3 antibodies that mediated antibody-dependent cellular cytotoxicity (ADCC), or antibody-dependent cellular phagocytosis (ADCP), correlated with a decreased risk of infection in the RV144 trial; however, these responses decreased more than 10-fold in the first 6 months after the vaccination [62,72]. Thus, a vaccine vector or regimen that elicits more durable Env IgG3 antibody responses is desirable. The durability of antibody responses was not assessed in this study; however, based on the results of Palli et al. [11], it is likely that an MVA or LSDV boost could elicit long lasting antibody responses.

Heterologous vaccine regimens often generate better immune responses than homologous regimens [33,36,72]. However, no obvious differences were observed in the antibody responses after two heterologous poxvirus boosts, when compared to the two homologous boosts in this experiment. Previous work, carried out by our group in macaques, demonstrated that the combination of MVA and LSDV generated broader, higher magnitude T cell responses than either vector alone [28]. However, these vaccines expressed a polyprotein of HIV-1 Gag, RT, Tat, and Nef (Grrtn) and no envelope; therefore, antibody responses to Env were not assessed. It is likely that the vaccine regimens described in this study elicited different cellular immune responses. Given that Gag-specific T cell responses have been shown to correlate with viraemic control [32], comparing this response would have been informative. Unfortunately, due to the limitation of the rabbit model, no suitable tools were available to assess T cell responses in this experiment.

Veterinary studies have shown the T cell response to LSDV to be critical for the protection against disease. A large proportion (approximately 50%) of vaccinated animals do not develop antibody responses, yet they are protected against LSD [16]. The interplay between humoral and T cell responses is not fully understood for LSDV, but both arms of the immune response are recognized as being important [27,73,74]. A balanced CD4^+^ and CD8^+^ T cell response specific to LSDV was elicited against a dual vaccine against LSDV and Rift Valley fever virus [75].

Improvements were made to the HIV-1 antigens used in LSDVGC5, compared to the LSDVgrrtn utilized in previous studies carried out by our group [28,29]. Grrtn was replaced with a subtype C mosaic ag which forms virus-like particles and a modified envelope gene was included. The binding and neutralizing antibody responses elicited against HIV-1 Env by the recombinant LSDVGC5 were comparable to those induced by MVAGC5 but did not show any significant advantages over previously used poxvirus vectors. Targeted sequence changes to the envelope could be carried out to improve the neutralizing antibody responses. Furthermore, more in-depth studies investigating the T and B cell responses elicited by the LSDV-vectored HIV vaccine in non-human primates or mice are warranted. These studies should include an assessment of both neutralizing and non-neutralizing antibody responses.
